# Clinical features and risk factors for community-onset bloodstream infections among coronavirus disease 2019 (COVID-19) patients

**DOI:** 10.1017/ice.2021.88

**Published:** 2021-03-12

**Authors:** Elisa F. Akagi, Mamta Sharma, Leonard B. Johnson, Susanna M. Szpunar, Kathleen Riederer, Louis D. Saravolatz, Ashish Bhargava

**Affiliations:** Ascension St John Hospital, Detroit, Michigan

Coronavirus disease 2019 (COVID-19) has caused millions of deaths around the world since it was declared a pandemic in 2020. A multicenter study observed that patients with bloodstream infections (BSIs) had more severe illness at the time of presentation and worse clinical outcomes.^1^ Increases in the demand for blood cultures (BCs) among patients presenting with fever puts a strain on available resources.^1^ Thus, it is essential to identify those patients who are at risk of BSI and could be at risk for poor outcomes. We analyzed the risk factors, present at the time of admission, for community-onset bloodstream infections (COBSIs) in COVID-19 patients.

## Methods

We performed a retrospective, single-center, case–control study at a 776-bed tertiary-care center. We included hospitalized patients with COVID-19 disease as confirmed by polymerase chain reaction (PCR) assay, who had a BC done on admission, between March 8 and June 14, 2020. COBSI was defined as a positive BC with a known pathogen in 1 or more BCs or the same commensal organism in 2 or more BCs drawn within 48 hours of hospitalization. Cases were classified as patients with confirmed COBSIs and controls had negative BCs upon hospital admission. Patients were excluded if a blood sample for culture was not drawn within the first 48 hours of hospitalization or if their positive BC was a contaminant. Data on clinical significance and the source of the BSI were collected from infectious diseases notes. Key epidemiological, demographic, clinical, laboratory, microbiologic, and outcome data were abstracted from the electronic medical record. Laboratory values were recorded from the date of admission. The study was approved by the institutional review board.

Statistical analyses were performed using SPSS version 27.0 software (IBM, Armonk, NY). Univariable analysis was done using the Student *t* test, the Mann-Whitney U test, and χ^2^ analysis. Predictors of mortality that were significant or near significant (*P <* .09) were entered in a multivariable logistic regression model using a forward likelihood ratio algorithm. For comorbidities, the Charlson weighted index of comorbidity (CWIC) score was used instead of individual comorbid conditions.^2^ Results from the regression are reported as odds ratios with 95% confidence intervals. All reported *P* values are 2-sided.

## Results

In total, 565 COVID-19 patients were admitted during the study period. Of these, 266 (47.1%) patients had a BC on admission. The mean age of the cohort was 64.6 (SD±16.4) years, 152 (57.1%) were male, and 206 (77.4%) were black. The mean body mass index of the cohort was 31.77 (±9.2) kg/m^2^. The common comorbid conditions were hypertension (75.2%), obesity (52.3%), and diabetes (36.8%). Also, 23 (8.6%) of 266 patients had a COBSI: 22 (95.7%) positive BCs were bacterial and 1 (4.3%) grew *Candida*. The common organisms isolated from the BCs were *Staphylococcus aureus* (34.7%) followed by *Escherichia coli* (13.0%), *Streptococcus pneumoniae* (8.7%), and *Enterococcus* spp (8.7%). The most common source was lung (34.8%), followed by soft-tissue infections (17.4%). Empiric antibiotic therapy was given for most cases (20 of 23, 87%).

The prevalence of COBSI was higher in males and patients admitted from nursing homes (Table 1). Patients with a COBSI were more likely to have dementia, hemiplegia, and moderate-to-severe liver disease than controls. Patients with a COBSI were more likely to present with altered mental status (AMS) but were less likely to have fever or shortness of breath. Cases were more likely to have leukopenia, low albumin and had lower mean hemoglobin than controls.

**Table 1.** Univariable Analysis of Factors Associated With Bacteremia on Admission Among COVID-19 Infected Patients

Characteristic	Negative BC (*n* = 243), No. (%)	Positive BC (*n* = 23), No. (%)	OR (95% CI)	*P* Value
**Age group**				
<70 y	146 (60.1)	14 (60.9)	1.0 (0.4–2.3)	.94
≥70 y	97 (39.9)	9 (39.1)		
**Sex, no. (%)**				
Male	134 (55.1)	18 (78.3)	2.9 (1.1–8.1)	.03
Female	109 (44.9)	5 (21.7)		
**Admission source**				
Home	179 (73.7)	9 (39.1)	4.4 (1.8–10.5)	.001
Nursing facility	64 (26.3)	14 (60.9)		
**Comorbidities, no. (%)**				
Myocardial infarction	19 (7.9)	3 (13.0)	1.7 (0.5–6.5)	.38
Congestive heart failure	32 (13.2)	5 (21.7)	1.8 (0.6–5.3)	.26
Cerebrovascular disease	35 (14.4)	4 (17.4)	1.3 (0.4–3.9)	.70
Dementia	34 (14.0)	8 (34.8)	3.2 (1.3–8.3)	.009
Chronic pulmonary disease	65 (26.7)	4 (17.4)	0.6 (0.2–1.8)	.33
Diabetes	89 (36.6)	9 (39.1)	1.1 (0.5–2.7)	.81
Hemiplegia	15 (6.2)	4 (17.4)	3.2 (1.0–10.6)	.046
Renal disease	46 (18.9)	5 (21.7)	1.2 (0.4–3.4)	.74
Mild liver disease	6 (2.5)	1 (4.3)	1.8 (0.2–15.6)	.59
Moderate-severe liver disease	1 (0.4)	1 (4.3)	11 (0.7–182.0)	.04
Median CWIC (IQR)	1 (0–2)	2 (2–4)		.001
Hypertension	182 (74.9)	18 (78.3)	1.2 (0.4–3.4)	.72
Morbid obesity	48 (20.2)	1 (4.3)	0.2 (0.02–1.4)	.06
Mean qSOFA score on admission	0.91 ± 0.74	1.5 ± 0.79		.001
**Symptoms, no. (%)**				
Altered mental status	65 (26.7)	13 (56.5)	3.6 (1.5–8.5)	.003
Fever	149 (61.6)	9 (39.1)	0.4 (0.2–1.0)	.04
Shortness of breath	184 (76.7)	11 (50.0)	0.30 (0.1–0.7)	.006
**Laboratory findings on admission, no. (%)**				
Leukopenia (<4 K/µL)	18 (7.4)	5 (21.7)	3.5 (1.2–10.4)	.02
Lymphocytopenia (<1K/µL)	130 (53.7)	13 (56.5)	1.1 (0.5–2.6)	.80
Thrombocytopenia (<150K/µL)	55 (22.7)	5 (21.7)	0.9 (0.3–2.7)	.91
Hemoglobin (gm/dL)	12.7 (±2.3)	10.6 (±3.0)		<.0001
Elevated ALT (>40 U/L)	81 (34.2)	9 (40.9)	1.3 (0.5–3.3)	.53
Low protein (<6.2 g/dL)	19 (7.9)	1 (4.5)	0.6 (0.07–4.3)	.57
Low albumin (<3.5 g/dL)	107 (44.8)	17 (77.3)	4.2 (1.5–11.7)	.003
Elevated CRP (>10 mg/L)	214 (93.9)	18 (94.7)	1.2 (0.1–9.5)	.88
Elevated procalcitonin (>0.10 ng/dL)	175 (79.9)	12 (80.0)	1.0 (0.3–3.7)	.99
Elevated creatinine (from baseline)	102 (44.3)	9 (42.9)	0.9 (0.4–2.3)	.90

Note. OR, odds ratio; CI, confidence interval; SD, standard deviation; CWIC, Charlson weighted index of comorbidity; IQR, interquartile range; ALT, alanine aminotransferase; CRP, C-reactive protein.

From multivariable logistic regression, 3 factors remained as predictors of COBSI: AMS (OR, 2.8; 95% CI, 1.05–7.4); admission from a nursing home (OR, 2.9; 95% CI, 1.1–7.7); and mean hemoglobin on admission (OR, 0.71; 95% CI, 0.59–0.86).

## Discussion

In our study, the prevalence of COBSI was 8.6%, which was higher than previously reported rates of 1.6%–2.5%.^1,3,4^ Previous studies did not report nursing home admissions. In our study, 30% of COVID-19 patients were admitted from the nursing homes. Admission from a nursing facility was an independent risk factor for BSI, likely due age-associated changes in immunity and multiple comorbidities in nursing home patients.^5^ In our study, a higher median CWIC was noted among COVID-19 patients with COBSIs than those with no COBSI, a finding consistent with a previous report among non–COVID-19 patients.^6^

AMS was independently associated with COBSI among COVID-19 patients. We previously reported higher mortality among COVID-19 patients who presented with AMS than those patients without AMS.^7^ A previous study with non–COVID-19 patients also reported that the severity of encephalopathy correlated with bacteremia.^8^

In our study, a lower mean hemoglobin was independently associated with COBSI. Possible mechanisms may be reduced oxygen saturation at infected sites and low hemoglobin as a marker of underlying conditions predisposing to infections.^9^

Our study has several limitations. First, as a single-center study, results may not be generalizable to other populations. In this study, 29% of the patients were admitted from nursing facilities. Second, in chart review studies, many patients may have missing data. Third, no systematic testing for coinfections was performed; blood cultures were ordered based on clinical suspicion for bacterial infection.

In conclusion, we observed a higher COBSI rate than previously reported. Admission from nursing facilities, altered mental status, and low hemoglobin at the time of hospitalization were independently associated with bacteremia on admission. Multicenter studies are necessary to validate the findings of our study.
